# The influence of music environment on conceptual design creativity

**DOI:** 10.3389/fpsyg.2023.1052257

**Published:** 2023-02-09

**Authors:** Tiansheng Xia, Yongqing Sun, Yi An, Linli Li

**Affiliations:** School of Art and Design, Guangdong University of Technology, Guangzhou, China

**Keywords:** music environment, creativity, design creativity, divergent thinking, convergent thinking

## Abstract

**Introduction:**

Creativity plays an important role in design. However, there have been mixed results about whether music, as an environmental stimulus, improves design creativity performance.

**Methods:**

Participants were 57 design major students who were randomly assigned to one of three groups, with 19 students in each group: no music, pure music, and music with intelligible semantic information (unrelated to the task) playing in the background. Each participant completed a design task (design a tool for storing painting materials), with two phases in it, one that involved idea generation (divergent thinking) and one that involved idea evaluation (convergent thinking). Performance in the two phases was rated based on six indices of creativity (fluency; flexibility; adaptability; feasibility; usefulness; novelty) and overall design creativity (ODC).

**Results:**

The results of one-way ANOVAs with Bonferroni correction showed that neither music environment had a significant influence on divergent thinking in idea generation nor convergent thinking in idea evaluation. However, both music environments had a significantly positive effect on novelty and ODC.

**Discussion:**

We discuss the implications of our current results for fostering designers’ creativity performance.

## 1. Introduction

In the 21^st^ century, creativity is considered one of the most important thinking skills (Ahmadi et al., 2019). Sarkar and Chakrabarti (2008) analyzed more than 160 definitions using majority analysis and relational analysis methods and proposed a “common” definition of creativity: creativity occurs through a process in which an agent uses its ability to generate novel and valuable ideas, solutions, or products.

Creativity is thought to include two measurable cognitive components: divergent and convergent thinking (Guilford et al., 1967). Divergent thinking refers to the process of generating creative ideas by proposing multiple solutions (Daikoku et al., 2021), which can reflect latent creativity (Runco and Acar, 2012). Convergent thinking is rule-based and conducive to analyzing cause-and-effect relationships between related items (Gabora, 2010), which emphasizes accuracy and logic and requires deductive reasoning to find the most appropriate solution (Jones et al., 2011; Lee and Therriault, 2013).

Divergent and convergent thinking work together to produce creative and valuable ideas (Sitorus and Masrayati., 2016). After exploring multiple possible solutions to a problem through divergent thinking, convergent thinking can be used to organize the structure of those solutions and help determine the best one. Thus, divergent thinking is essential for concept generation (Yilmaz and Daly, 2016), whereas convergent thinking is indispensable for articulating various solutions as well as identifying creative and valuable ideas (Goldschmidt, 2016).

### 1.1. Creativity and the music environment

Individual creativity may be influenced by a variety of factors, such as the work environment, stimulation received in the creative process, and interaction with other individuals (Han et al., 2021). Researchers have found that creative performance can be mediated by the interaction between creative processes and the work environment (Hunter et al., 2007). The work environment (García-García et al., 2019; Chulvi et al., 2020b; Daikoku et al., 2021; Fleury et al., 2021) has been shown to affect creativity. Among these work environment variables, music has been considered an environmental stimulus that can affect individual creativity (Hickey, 2012; Webster, 2012). For example, it has been shown to affect divergent thinking, convergent thinking, and other creativity task performance (Ritter and Ferguson, 2017; Threadgold et al., 2019).

Some researchers have found that music plays a positive role in promoting individual creativity (Ritter and Ferguson, 2017; Zhou et al., 2020). Ritter and Ferguson (2017) explored four types of music (happy, sad, calm, and anxious) as well as the effects of different affective valence (positive, negative) and arousal (high, low) on individual creativity. Listening to “happy music” (high-arousal classical music that can elicit positive emotions) was more conducive to divergent thinking than the control condition (no music). This may be due to the influence of emotion and situational variables on the persistence and flexibility of creative activities (Nijstad et al., 2010). Flexibility is the ability to switch between stimuli, operations, and mental settings in response to demands (Miyake et al., 2000; Vartanian, 2009). That is, when people get into difficulties in viewpoint exploration, emotion and situation variables help people to think differently to solve more problems. Functional magnetic resonance imaging (fMRI) analysis also showed that positive emotional states increased preparatory activity in the anterior cingulate cortex and engaged participants in problem processing, which facilitates problem-solving with insight (Subramaniam et al., 2009).

By contrast, convergent thinking helps with finding a correct answer, which requires less fluency and flexibility (Goldschmidt, 2016). Therefore, a “happy” music environment does not have a significant impact on the performance of tasks related to convergent thinking (Ritter and Ferguson, 2017). Another study explored the effect of music with different rhythms on creative performance (Zhou et al., 2020). Participants completed a series of “30-lap tests” designed to measure creativity in a quiet environment or an environment with music (fast or slow). The fast-paced music environment significantly improved the creative performance of novice designers in terms of fluency and flexibility (related to divergent thinking; Zhou et al., 2020). It was shown in another study that people’s design creativity increases significantly in arousing environments (García-García et al., 2019). Music may have an impact on individual creativity through the indirect (mediating) effect of emotional response (He et al., 2017). Fast-paced music increases arousal, which may in turn promote creative performance (He et al., 2017).

However, other studies have suggested that music or background sounds may impair performance on creative tasks (Threadgold et al., 2019; Marshalsey, 2021). A recent study investigated the impact of different types of musical environments on the performance of Composite Remote Association Tasks (CRATs; Threadgold et al., 2019). The CRATs is considered to be an effective test of creativity, and high performance on this test is associated with convergent thinking (Bowden and Jung-Beeman, 2003). The results showed that compared with exposure to a quiet environment, exposure to music (regardless of whether the music was familiar or unfamiliar, with or without lyrics) had a greater inhibitory effect on creative performance (Threadgold et al., 2019). This result challenges the idea that the musical environment enhances creativity (Ritter and Ferguson, 2017). The short-term visual-verbal serial recall task is often used to test cognitive interference caused by ignored background sounds (Colle and Welsh, 1976; Salamé and Baddeley, 1989; Jones and Macken, 1993). Some researchers assert that music is a changing state of sound (rather than a steady-state sound) that impairs serial recall, thus inhibiting the process of verbal working memory and negatively affecting insight and problem-solving (Salamé and Baddeley, 1989; Schlittmeier et al., 2008; Threadgold et al., 2019). Researchers have found that music with lyrics or sounds with comprehensible semantics tends to interfere with understanding cognitive tasks (Martin et al., 1988; Braat-Eggen et al., 2017). This idea was also tested in a recent study (Marsh et al., 2021) in which researchers manipulated the intelligibility of auditory materials in background music, and the results showed that the presence of intelligible semantic information (unrelated to the task) in background music impaired participants’ performance in CRATs, compared the control group.

### 1.2. Problems with existing research

We compared these studies and found that music promoted creativity mostly in terms of divergent thinking. In contrast, studies that found that music interfered with creativity mostly used convergent thinking as the index of creativity. Marsh et al. (2021) used the CRAT as a creativity task and concluded that music with understandable semantics interfered with creative performance. Ritter and Ferguson (2017) used the Alternative Use Task (AUT) to assess the effects of music on divergent thinking and used the Remote Association Task (RAT) to evaluate convergent thinking respectively, the results found that listening to “happy” music can increase divergent thinking, but not convergent thinking. In addition, these studies often assessed the influence of the musical environment on creativity by focusing on one aspect of the creative process (divergent or convergent thinking) or assessing these two aspects with different tasks separately. In reality, the creative process involves both idea generation and idea evaluation process (Harvey, 2013), it includes both convergent and divergent thinking (Guilford et al., 1967; Brophy, 1998; Ichino, 2011; Goldschmidt, 2016; Webb et al., 2017). Besides, The AUT and RAT are mainly used to measure the thinking component of creativity, which is abstract and general. Moreover, these two assessment methods also have some limitations.

For example, an AUT test score may be affected by some nonsense words (Hass, 2017; Forthmann et al., 2019), whereas the RAT may be influenced by the participants’ linguistic background and verbal ability (Becker and Cabeza, 2022). Behrens and Olteţeanu (2020) reported some differences between RAT tests in different languages; however, it is not clear whether these differences are due to problem-solving ability. In addition, for individuals with limited vocabularies, language-dependent RAT tests are not suitable for evaluating creative abilities (Becker and Cabeza, 2022). These limitations may lead to differences between the measured scores and the actual use of creativity.

### 1.3. Design creativity task

Therefore, our research introduces a more ecologically valid creativity task, namely a design creativity task. Design creativity refers to the ability to produce novel designs (Dumas et al., 2016), which is an embodiment of creativity in the field of design. Design activity is the process of exploring problem and solution spaces to find a particularly favorable solution. The design process can be divided into routine and non-routine designs (Gero, 2000). Routine design can be defined as the design activities that occur when all necessary knowledge is available, while non-routine design can be subdivided into innovative and creative designs, which produce different effects: the design process and the design result of a product or artifact, respectively (Gero, 2000). Because the problem-solving situations involved in design activities are usually characterized by unclear definitions and high openness (Casakin and Kreitler, 2011), problems often cannot be solved by applying routine problem-solving procedures (Gero, 2000). In previous studies, general creativity tasks were mostly used to examine the relationship between music and creativity rather than a realistic problem-oriented non-routine design creativity task (Sarkar and Chakrabarti, 2011).

Different from previous general creativity tasks, the design creativity task includes two distinct aspects: creativity in the process of product design, and creativity in the design result. Design involves divergent and convergent thinking to identify the core of a problem, to then devise a particularly favorable solution for that challenge (Dorst and Cross, 2001; Liu et al., 2003; Liang et al., 2019). Creativity in the process of product design is related to divergent thinking while creativity in the design result is related to convergent thinking (Webb et al., 2017). In terms of process, prior research has emphasized the rational decision to find design solutions in the “problem-solving process,” and the creativity of design results is often studied to evaluate the novelty and usefulness of product design (Sarkar and Chakrabarti, 2011). Some researchers assert that music contributes significantly to the creative generation; for example, music may help to stimulate special associations (Liao and Chang, 2015). Then, what kind of influence does music have on creativity in the design process and the innovativeness of design works in a design activity that includes divergent and convergent thinking? That is the question the current study will address. To analyze the creative performance in a different phase of conceptual design under different musical environments, three groups of participants were compared. In the evaluation phase, we evaluated the scheme from three indices: divergent thinking, convergent thinking, and overall design creativity (ODC). Among them, fluency and flexibility are indicators related to divergent thinking, while adaptability and feasibility are indicators related to convergent thinking (Bonnardel and Didier, 2020). According to the “common definition of creativity” proposed by Sarkar and Chakrabarti (2008), creativity should be measured directly in terms of novelty and usefulness (Sarkar and Chakrabarti, 2011).

### 1.4. Aims and hypothesis

This study aimed to explore whether the musical environment affects designers’ creativity during the conceptual design phase. Previous research has shown that musical background affects creative performance (Bowden and Jung-Beeman, 2003; Marsh et al., 2021). As mentioned above, the creative tasks in studies where music promoted creative performance were usually divergent thinking tasks (e.g., AUT), so we assumed that music in the background would promote divergent thinking. In addition, studies that have found that musical background inhibits creative performance usually used convergent thinking tasks (e.g., RAT/CRAT). We hypothesize that musical background inhibits convergent thinking. Combined with the inhibitory effect of semantic interference on convergent thinking, we further deduced that music with an intelligible semantic background has a more obvious inhibitory effect on convergent thinking. Creative processes involve both divergent and convergent thinking; therefore, the effect of musical background on overall creativity remains unclear. Given the null results of the inhibitory effect on convergent thinking from a music background in previous research (Ritter and Ferguson, 2017), novelty is likely to be related to divergent thinking (Diedrich et al., 2015), which is an important index of overall creativity. Therefore, we hypothesized that musical background may promote overall creativity. Our hypotheses were as follows:

*H1*: Both pure music and intelligible semantic music background have a positive effect on divergent thinking (fluency and flexibility).

*H2*: Both pure music and intelligible semantic music background have a negative effect on convergent thinking (adaptability and feasibility), and music with intelligible semantics has a stronger effect.

*H3*: Both pure music and intelligible semantic music backgrounds have a positive impact on ODC (novelty, usefulness, and the product of them).

## 2. Methods

### 2.1. Participants

We recruited 60 industrial design and product design students (32 females; 28 males) with a mean age of 21.6 years (*SD* = 1.02) to participate. Since they were students majoring in design, they constitute an ideal sample for this research. They were informed of the overall goal of the experiment and agreed in writing to participate. The participants were paid CNY 30 at the end of the experiment. All participants spoke Chinese as a first language and reported normal vision (or normal corrected vision) and normal hearing.

### 2.2. Experimental design, stimuli, and procedures

A single-factor, three-level (pure music, semantically intelligible background music, and no music) between-subject design was used. Participants were randomly assigned to one of three groups, each with 20 participants. The stimuli material of pure music is *The 4 Seasons, Op. 8, No. 1, RV 269, SpringdMvt 1. Allegro* (Ritter and Ferguson, 2017). Szalma and Hancock (2011) found that phonological types of noise are the most damaging to human performance during cognitive tasks. Therefore, the semantically intelligible background music group listened to a combination of Li Bai’s poetry recitation and background music, as this is a semantically intelligible changing-state sound, and poems by Li Bai (one of the most famous poets in China) are familiar to Chinese students. In the control group, participants completed the design task in a quiet environment. The dependent measures were fluency, flexibility, adaptability, feasibility, usefulness, novelty, and ODC (Table 1).

**Table 1:** Different music stimuli.

Group	Music materials
1. Pure music	*The 4 Seasons, Op. 8, No. 1, RV 269, SpringdMvt 1. Allegro*
2. Intelligible semantic music	*Poetry recitation with background music from The 4 Seasons, Op. 8, No. 1, RV 269, SpringdMvt 1. Allegro*
3. Control group	No music

The lab is equipped with a desk and an office chair. In the experiment, Sony WI-1000X wireless Bluetooth noise canceling headphones produced by Sony Ltd., Japan, were used to play music materials for participants. Participants were given the same materials including unlimited amounts of A4 paper, hard crayons, markers, pencils, sharpeners, and erasers. The graphic quality of participants’ drawing schemes was not taken into account in this study. Participants were asked to engage in a design task with two creative phases and propose conceptual design solutions related to the task. To make it easy to obtain multiple solutions, the description of the design tasks was quite open, with few restrictions.

**Figure 1:**
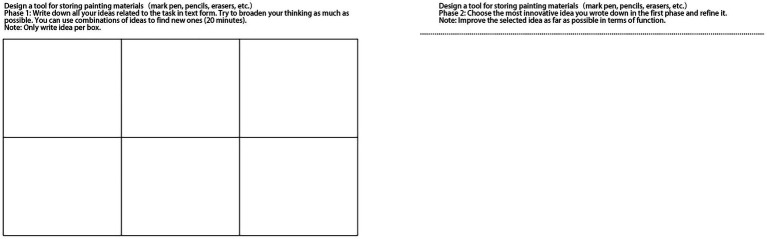
Design task. For phase 1, participates have 20 min to design a tool for storing painting materials. They were told to draw out as many of their ideas as possible. For phase 2, participants were asked to choose the most innovative idea and then refine it by sketching it out. Participates had 20 min for this phase.

Participants all received the same design task description: design a tool for storing painting materials (Chulvi et al., 2020a; see Figure 1 for an example), and they were told to draw out as many of their ideas as possible. The experiment was divided into two phases, and each phase had 20 min to finish. In the first phase (divergent thinking phase), participants were given 20 min to read the task introduction and the conditional rules and write down all their thoughts about the design task as most as they can. In the second phase (convergent thinking), participants were asked to choose the most innovative idea and then refine it by sketching it. The participants were given 20 min for this phase. In the selection process, participants need to consider novelty and choose the most innovative idea. In the process of sketching, they must consider the usefulness and improve their selected idea as much as possible in terms of function. We provided these instructions during the experiment to ensure that participants understood the task expectations. The participants were asked to wear headphones throughout the session and complete the design task with the appropriate music in the background (no music was played in the control group).

**Figure 2:**
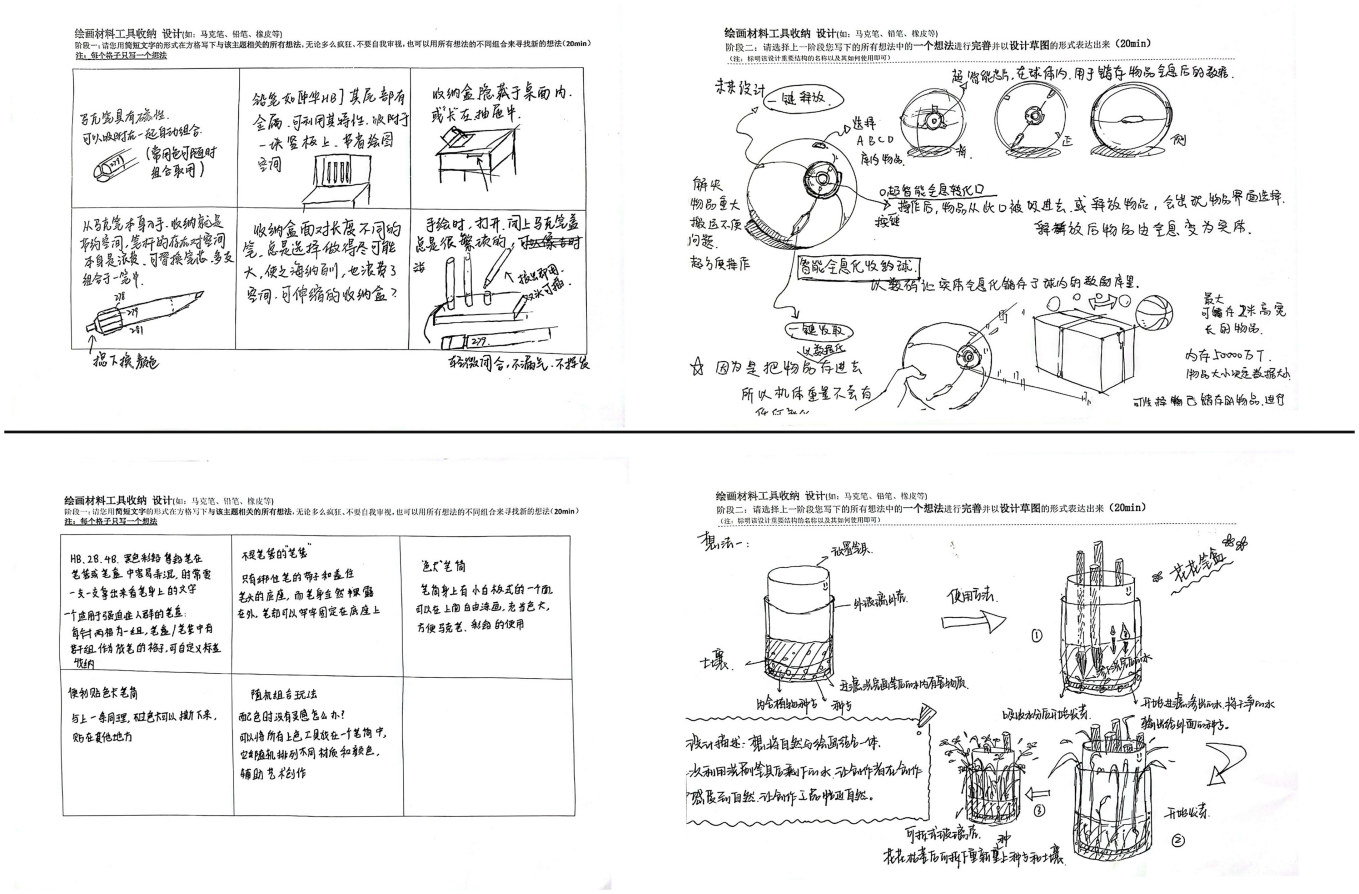
Examples of solutions.

There were 57 participants who completed the design task and three samples were eliminated because of the incomplete design scheme. Participants created the design and obtained different conceptual design solutions (please see Figure 2 for an example). We recruited three postgraduate design students to evaluate the design projects of all participants in the three different music environments and to rate the creativity of the design results provided by the participants.

### 2.3. Methods of the evaluation

#### 2.3.1. Evaluation criteria for divergent thinking

Divergent thinking is the ability to generate a variety of possible answers or different solutions to a problem and is marked by fluency, flexibility, and originality (Kim, 2006; Kharkhurin, 2017). In our experiment, fluency and flexibility were selected as the measures of divergent thinking. Fluency is a measure of creative production, determined by the total number of creative ideas produced. The fluency score requires counting the total number of complete ideas (no unfinished ideas) listed by the participant. Flexibility is manifested in different cognitive categories and perspectives, which can be measured by the number of different thought categories represented by the participants’ ideas (Ritter and Ferguson, 2017). According to Shah et al.’s measurement of diversity categories (Shah et al., 2003), we examined how each function is satisfied in terms of four dimensions (i.e., physical principle, working principle, embodiment, and detail) and assigned these dimensions weights of 10, 6, 3, and 1, respectively. Weights were chosen to provide a more meaningful classification when achieving higher overall scores. If a given category has only one dimension, no diversity is displayed, and the score should be zero; otherwise, the score should be multiplied by the number of dimensions by the corresponding weight. The raters then calculated the sum score for each measure of divergent thinking.

#### 2.3.2. Evaluation criteria for convergent thinking

The Convergent Thinking Test measures whether participants successfully come up with the most suitable answer (Clements-Croome, 2006). In this experiment, adaptability and feasibility were used as measures of convergent thinking, as in other research (Bonnardel and Didier, 2020). Adaptability was determined by the relevance of the final solution and the design task. Raters evaluated adaptability on a Likert scale from (1 = completely irrelevant) to (5 = very relevant). The feasibility of the final solution was evaluated in terms of economic feasibility, technical feasibility, social feasibility, and other forms of feasibility. Raters evaluated all these components of feasibility using a Likert scale from (1 = not feasible at all) to (5 = very feasible).

#### 2.3.3. Evaluation criteria for overall design creativity

The ODC scores for the participants’ solutions were calculated using the Moss metric, based on the product of the original scores for novelty and usefulness (Moss, 1966). This method has good reliability and validity and is widely accepted by many researchers (Sarkar and Chakrabarti, 2011; Chulvi et al., 2012, 2020a,b; Toh and Miller, 2016). Chulvi et al. (2020a,b) evaluated the design creativity performance of product designers in different environments using the Moss metric and confirmed that this method was well-suited for capturing product creativity in the context of studies examining the impact of environment on creative design performance, similar to our study design.

First, Sternberg and Lubart (1999) define novelty as being “not similar to something previously known.” Thus, novelty can be measured as the inverse probability that an idea appears in a homogeneous set of solutions. The frequencies of the proposed scheme concepts were reported as percentages: (0 = very common, >10% of similar concepts), (1 = relatively common, 5–10%), (2 = relatively rare, <5%), and (3 = very rare or original concepts, 1%). All three design graduate raters had sound professional foundations and competence and had evaluated the same design topic in similar experiments many times to ensure they were familiar with possible solutions.

Second, usefulness was determined by raters’ evaluations of the degree to which the functional requirements of the product conform to the standard solution, which ensures the quality of product design while accomplishing the basic functions. The degree of functional perfection of the participants’ solution was evaluated as belonging to one of four categories: 0 = does not fulfill the basic function, 1 = only fulfills the basic function, 2 = reaches the level of quality of the standard solution, and 3 = the solution is better than the standard one at the functional level. Finally, ODC was obtained by multiplying these two variables, with a final score between 0 and 9, calculated as creativity = usefulness × novelty.

### 2.4. Data analysis

All statistical analyses were performed using SPSS version 23 (IBM Corp. NY, United States, Armonk). The fluency, adaptability, and overall design creativity were skewed (|2 × SE SKEW| < Skewness statistics). Skewness was corrected by applying a square-root transformation. The correlations between the transformed and untransformed values were high (>0.95), indicating that the transformation of values did not change the general interpretation of the findings. ANOVAs were conducted on the transformed data, and the Bonferroni correction was used. Descriptive statistics of the non-transformed data are mentioned.

ANOVA was used to test for significant group differences in the three measures of creativity (convergent, divergent, and ODC) represented by seven subscale scores (fluency, flexibility, adaptability, feasibility, usefulness, and novelty). Significance values were adjusted using the Bonferroni correction for the three measures. The inter-rater reliability of the ratings was calculated using a 2-way random intraclass correlation coefficient (ICC) analysis for consistency, ICC_fluency_ = 0.884, ICC_flexibility_ = 0.660, ICC_adaptability_ = 0.537, ICC_feasibility_ = 0.574. The three experts discussed the values of novelty, usefulness, and ODC, with each parameter providing an average score for statistical analysis (Chulvi et al., 2017).

## 3. Results

The raters’ evaluations of multiple indices of creativity and ODC in the pure music, intelligible semantic, and control groups are shown in Table 2.

**Table 2:** Means and standard deviations of the assessment of creativity factors.

	Pure music		Intelligible semantic music		Control group		*F*	*p*
	*M*	*SD*	*M*	*SD*	*M*	*SD*		
Fluency	11.11	6.60	10.32	7.14	8.68	4.75	0.74	0.481
Flexibility	4.52	1.43	4.00	1.77	3.91	1.15	0.97	0.387
Adaptability	4.21	1.04	3.70	0.87	4.26	0.50	2.63	0.081
Feasibility	3.54	0.98	2.82	0.91	3.33	0.87	3.09	0.054
Usefulness	1.47	0.61	1.26	0.56	1.21	0.42	1.28	0.287
Novelty	1.37	1.16	1.53	1.02	0.42	0.61	7.36	0.001**
ODC	2.16	1.17	2.05	1.77	0.45	0.62	6.57	0.003**

ODC, overall design creativity, *n* = 19 (each group).

** *p* < 0.01.

### 3.1. Divergent thinking

In terms of fluency, the ANOVA showed no significant difference across the three groups. The group averages are reported as Means and Standard Deviations: pure music group (*M* = 11.11 ± 6.60), intelligible semantic music group (*M* = 10.32 ± 7.14), and control group (*M* = 8.68 ± 4.75), *F*(2, 54) = 0.74, *p* = 0.481. The results showed that the two musical environments had no significant effect on fluency in divergent thinking compared with the control condition.

As for flexibility, the ANOVA showed that there was no significant difference between the pure music group (*M* = 4.52 ± 1.43), intelligible semantic music group (*M* = 4.00 ± 1.77), and control group (*M* = 3.91 ± 1.15), *F*(2, 54) = 0.97, *p* = 0.387. The results showed no differences in flexibility in divergent thinking among the three groups.

### 3.2. Convergent thinking

In terms of adaptability, the ANOVA showed no significant difference among the pure music group (*M* = 4.21 ± 1.04), intelligible semantic music group (*M* = 3.70 ± 0.87), and control group (*M* = 4.26 ± 0.50), *F*(2, 54) = 2.63, *p* = 0.081, and no *post hoc* comparisons were conducted. The results indicated no differences in adaptability in convergent thinking among the three groups.

Regarding feasibility, the ANOVA showed that there was no significant difference between the pure music group (*M* = 3.54 ± 0.98), intelligible semantic music group (*M* = 2.82 ± 0.91), and control group (*M* = 3.33 ± 0.87), *F*(2,54) = 3.09, *p* = 0.054, this difference was not significant; thus, no *post hoc* comparisons were conducted. Both the pure music and intelligible semantic music environments had no significant inhibitory effect on the feasibility dimension of convergent thinking.

### 3.3. Overall design creativity

Regarding usefulness, the ANOVA showed that there was no significant difference in usefulness between the pure music group (*M* = 1.47 ± 0.61), intelligible semantic music group (*M* = 1.26 ± 0.56), and control group (*M* = 1.21 ± 0.42), *F* (2, 54) = 1.28, *p* = 0.287, and no *post hoc* comparisons were conducted.

In the aspect of novelty, ANOVA showed that there were significant differences across the pure music group (*M* = 1.37 ± 1.16), intelligible semantic music group (*M* = 1.53 ± 1.02), and control group (*M* = 0.42 ± 0.61), *F*(2, 54) = 7.36, *p* = 0.001. *Post hoc* comparisons with Bonferroni correction revealed a significant difference between the pure music group and the control group (*p* = 0.004); a significant difference between the intelligible semantic music group and the control group (*p* = 0.001); and no significant difference between the pure music group and the intelligible semantic music group (*p* = 0.614). The results showed that compared with the control condition, both music environments appeared to promote novelty.

In terms of overall design creativity, the variance results showed that there were significant differences in design creativity among the pure music group (*M* = 2.16 ± 1.17), intelligible semantic music group (*M* = 2.05 ± 1.77), and control group (*M* = 0.45 ± 0.62), *F*(2, 54) = 6.57, *p* = 0.003. *Post hoc* comparisons with Bonferroni correction showed a significant difference between the pure music group and the control group (*p* = 0.006); the difference between the intelligible semantic music group and the control group was significant (*p* = 0.011), and the difference between the pure music group and the intelligible semantic music group was not significant (*p* = 0.845). The results showed that, compared with the control condition, the two kinds of music environment had a promotion effect on design creativity.

## 4. Discussion

The finding that listening to music promotes overall design creativity (ODC) is consistent with a previous study (Mustofa and Hidayah, 2020). Studies have shown that the working environment has an important impact on individual creative performance, and music in the background environment can promote emotional arousal (He et al., 2017), thus promoting the novelty of overall creativity, which is consistent with our results. However, in terms of usefulness, our results were not significant, and we speculate that this finding may be related to the characteristics of novelty and usefulness. Novelty is considered the main or even central characteristic of creativity, while usefulness is secondary (Amabile, 1988; Plucker et al., 2004; Storme and Lubart, 2012). A previous study showed that novelty can be regarded as a first-order criterion of creativity, while usefulness is a second-order criterion of creativity. This means that usefulness will be shown in actual creativity when an idea is novel, whereas usefulness is not important if an idea is not novel (Diedrich et al., 2015). In a study on the impact of the environment on creativity, researchers also used novelty and usefulness as factors to evaluate creativity and found that usefulness was not affected by the environment (Chulvi et al., 2020b). A similar result was obtained in our study, which indicates that the environment may have little effect on usefulness.

Therefore, novelty can be used as a direct measure of whether creative performance is enhanced by musical background. Besides, our results may also be related to emotion. The music selected in this experiment was high-valence and high-arousal music (Ritter and Ferguson, 2017), which can induce positive emotions, thus facilitating the solution of design problems (García-García et al., 2019).

Unexpectedly, neither fluency nor flexibility had a significant effect on divergent thinking compared to the control condition. This result is at odds with our assumptions that two types of music (pure music; intelligible semantics music) would have a positive effect on the divergent thinking process. The difference in results across studies is most likely due to the difference between the AUT, used in other studies, and our design task. The AUT requires participants to consider as many uses as possible for simple everyday objects (such as bricks or paper clips; Hommel et al., 2011). A key feature of design activity, as used in the current study, is that designers get a brief description of what they will design (usually its general functionality and some limitations). For example, our participants were asked to design a tool to store painting materials, a task that is more restrictive than the AUT. However, fluency is calculated as the total number of ideas listed by participants (Zhu et al., 2019). Flexibility is measured by the number of different idea categories used by participants (Ritter and Ferguson, 2017) and it needs rich diversity of different ideas. However, the design task we used in the current study might narrow the direction and scope of the participants’ thinking, so there is less of an impact on flexibility and fluency.

Additionally, this result may be attributed to the research sample. The participants in the present study were all college students majoring in design, and design training may improve divergent thinking to a certain extent (Xia et al., 2021), so the average level of divergent thinking of participants in the present study may be higher than that of the general population, resulting in a null effect from the music environment.

In addition, the difference in convergent thinking was not significant, which is inconsistent with our hypothesis and previous findings (Threadgold et al., 2019; Marsh et al., 2021). This phenomenon may be related to the experimental stimulus material. Semantic processing is likely influenced by higher levels of cognition (Vachon et al., 2020). Li Bai’s poems are indeed familiar to Chinese students, which is likely to trigger the participants’ occasional unconscious automatic semantic processing, thus leading to a negative effect on semantic fluency. Studies have shown that the lower the frequency of speech interference, the greater the damage is to semantic fluency (Marsh et al., 2017). However, the design creativity task used in the present study is a problem-solving task, which requires less semantic processing than the RAT, so their impact on convergent thinking may not be obvious. Remarkably, the stronger facilitating impact of music on divergent and convergent thinking depended on the chosen music style. We used classical music as our experimental material, while other types of music, such as techno music (fast music), might have an even stronger enhancing effect on creativity (Gerra et al., 1998). Overall, the impact of music can be quite diverse, depending on the subjective music preferences of individuals and the type of music being played. In our experiment, the three dimensions measured (divergent thinking, convergent thinking, and ODC) belonged to different phases of the design process. Divergent thinking is generated in the conceptual design stage, convergent thinking is generated in the scheme creation stage, and the overall design creativity is determined by the presentation of the final design. In our design creativity task, the music background played a role in promoting the final presentation effect of product design, while the influence was not obvious during the phases of convergent and divergent thinking. The results may also be because the design task of the divergent thinking phase was different from that of the convergent thinking phase. The divergent phase consisted of a verbal task (writing down solution ideas), whereas the convergent thinking phase consisted of a visual task (drawing the selected idea). Therefore, any potential impact of music and semantic content could not be related to divergent versus convergent thinking because the conditions are confounded by verbal versus visual information processing (McKim, 1972).

The main limitation of this study is related to the participants’ characteristics. The number of participants was relatively small, and all students were majoring in industrial and product design. However, it is unclear whether these findings apply to all groups of professional designers. Another shortcoming is that we did not measure psychological variables or participants’ subjective appraisals during the experiment. Music can affect creative performance by inducing different emotions (Ritter and Ferguson, 2017), and people have quite different emotional reactions to the same piece of music (Gerra et al., 1998; Brattico and Jacobsen, 2009), so we could not effectively examine the psychological processes by which high-arousal music induces positive emotions to promote creative performance. Future studies should measure other psychological variables, as well as participants’ subjective appraisals of music, to further explore the possible influence of other factors. In addition, in the evaluation of novelty, occurrence frequency was evaluated based on the raters’ intuition. Future research can explore more accurate measurement methods for more rigorous evaluation. Finally, in our experiment, we chose classical music with high valence and arousal as the stimulus materials. In future studies, other types of music used in practice to enhance creativity can be chosen for more thorough analysis.

## 5. Conclusion

In this experiment, we manipulated environmental conditions (pure music, intelligible semantic music, and no music) and used a design creativity task to investigate the effect of the music environment on multiple indices of design creativity and overall creativity. The results showed that, compared with the control group, the two music environments significantly improved ODC, but only in the novelty dimension. Listening to either type of music did not effectively promote divergent thinking or impair convergent thinking.

## Data availability statement

The raw data supporting the conclusions of this article will be made available by the authors, without undue reservation.

## Ethics statement

Studies involving human participants were reviewed and approved by the Academic Ethics Committee of the Guangdong University of Technology. All participants provided written informed consent to participate in the study.

## Author contributions

TX and LL designed the study. YS and TX collected and analyzed the data. TX, YS, and YA wrote the first draft of the manuscript and revised the manuscript. All authors contributed to the article and approved the submitted version.

## Funding

This research was funded by grants from the National Social Science Foundation of China (18BYY089), the Young Scholar of Humanity and Social Science Grants from the Ministry of Education of the People’s Republic of China (20YJC760044), and the Higher Education Young Scholar Innovative Programs of Guangdong Province (2018WQNCX022).

## Conflict of interest

The authors declare that the research was conducted in the absence of any commercial or financial relationships that could be construed as a potential conflict of interest.

## Publisher’s note

All claims expressed in this article are solely those of the authors and do not necessarily represent those of their affiliated organizations, or those of the publisher, the editors and the reviewers. Any product that may be evaluated in this article, or claim that may be made by its manufacturer, is not guaranteed or endorsed by the publisher.
